# Bioprospecting Bacteria From Psychrophilic Anaerobic Digestate for Potential Plant Growth-Promoting Attributes

**DOI:** 10.1155/ijm/2208124

**Published:** 2025-04-23

**Authors:** Muyiwa Ajoke Akindolire, Busiswa Ndaba, Maryam Bello-Akinosho, Haripriya Rama, Ashira Roopnarain

**Affiliations:** ^1^Microbiology and Environmental Biotechnology Research Group, Agricultural Research Council-Natural Resources and Engineering, Pretoria, South Africa; ^2^Institute for Catalysis and Energy Solutions, College of Science, Engineering and Technology, University of South Africa-Florida Campus, Florida, South Africa; ^3^Department of Biological Sciences, Faculty of Natural and Applied Sciences, Summit University, Offa, Nigeria; ^4^Department of Environmental Sciences, College of Agriculture and Environmental Sciences, University of South Africa, Roodepoort, South Africa

**Keywords:** anaerobic digestion, cold-tolerant bacteria, plant growth-promoting bacteria, psychrophilic digestate, psychrotrophic bacteria

## Abstract

The psychrophilic anaerobic digestion (PAD) system is a diverse and underexplored microbial ecosystem that typically harbors cold-adapted microorganisms with possible agronomic potential. The plant growth-promoting bacteria in the residue of PAD have the potential to enhance crop production, particularly during cold winter months. In this context, the characteristics of cultivable, cold-tolerant bacteria isolated from digestate obtained during PAD were investigated. Of the 20 isolates, 12 (60%) were able to solubilize phosphate from insoluble compounds at 15°C. Furthermore, nine (45%) and six (30%) isolates exhibited nitrogen fixation activity and produced indole acetic acid (IAA), respectively, while only two (10%) isolates were capable of producing siderophores. Hydrolytic enzyme production varied with cellulase production observed as a common trait since all isolates produced varying levels of cellulase ranging from 3.3 ± 0.5 to 15.3 ± 4 mm activity diameter. Isolates *Comamonas* sp._A3-1, *Acinetobacter iwoffi*_B5-1, and *Pseudomonas* sp._B5-5 displayed maximum cellulolytic activity with activity diameters of 13 ± 2, 13 ± 1.2, and 15.3 ± 4 mm, respectively. However, only two (10%) of the bacterial isolates produced protease with *Pseudomonas* sp._B5-5 demonstrating maximum proteolytic activity as depicted by an activity diameter of 11.3 ± 2.5 mm. Nucleotide sequence analysis of seven isolates, possessing multiple plant-beneficial traits, revealed their affiliation to three genera: *Acinetobacter* (57%), *Comamonas* (28.7%), and *Pseudomonas* (14%). Biolog Phenotype MicroArray plates revealed varied catabolic capability among bacterial strains, with isolate B5-5 demonstrating the highest metabolic diversity. The findings of this study revealed that cold-tolerant isolates from low-temperature AD possess promising plant growth-promoting characteristics, which indicates the potential of psychrophilic digestate for application in agriculture.

## 1. Introduction

Psychrophilic and psychrotrophic microorganisms represent peculiar groups of cold-loving extremophiles with an inherent ability to survive and colonize low-temperature environments that would otherwise be lethal to other microbial life [1, 2]. While both categories of cold-adaptive microorganisms can grow at or below 15°C, psychrophiles can thrive at lower temperature zones compared to psychrotrophs and are therefore prevalent in permanently cold environments such as polar ice, glaciers, and deep oceans [3–5]. By contrast, psychrotrophic microbes, with an optimal growth temperature of 20°C or higher, exhibit a wide temperature range required for survival in cold habitats characterized by large seasonal temperature variations. As a result, they typically occur in temporarily cold environments such as wetlands, river sediments, landfills, and psychrophilic anaerobic digesters. However, they are also prevalent in permanently cold habitats [4, 6].

In addition to freezing temperatures, cold-adapted microbes must contend with other environmental stressors, and depending on the type of cold environment, this can vary from high pressure to low nutrient levels or high salinity and oxidative stress [7]. Consequently, cold-adapted microorganisms have developed various important physiological and adaptive strategies to overcome the challenges associated with low temperatures. Common mechanisms of thermal adaptation include the modification of membrane fatty acids for increased membrane fluidity at low temperatures and the production of antifreeze and ice-nucleating proteins as well as extracellular polymeric substances [1]. Other approaches involve the production of cold-active enzymes and the adjustment of metabolism to promote energy conservation and survival [1]. With these diverse arrays of survival strategies, they can maintain viability and even thrive in otherwise uninhabitable and harsh conditions. As such, this group of microorganisms is of particular interest given their great potential in low-temperature biotechnological processes including bioremediation, biodegradation of recalcitrant compounds, and the production of a variety of cold-active enzymes [8].

Of particular relevance is the potential application of cold-tolerant microorganisms in anaerobic digestion (AD) in cold regions. Low temperature constitutes a serious impediment to AD performance and is often cited as a key hindrance to the wider adoption of the technology in cold areas. Under the mesophilic range (30°C–40°C), AD performance is generally optimal; however, a decrease in temperature during winter or in cold climatic regions contributes to low biogas production and unstable operational performance of the AD system [9, 10]. Low temperatures adversely affect AD performance through reduced metabolism of the microbial community and subsequent effects on fermentation efficiency, as well as important process parameters [10, 11]. This ultimately leads to collapse of digesters in regions that often experience extremely low temperatures during winter seasons.

For instance, dramatic seasonal temperature variations occur in some parts of Europe, Asia, and Africa. These temperatures are often ≤ 20°C in over 3 months of the year. In addition, some regions in Europe, the United States, Canada, and Australia are generally more adapted for psychrophilic conditions [12, 13]. Therefore, measures to maintain AD under these conditions must be taken into consideration. Temperature changes can have an impact on AD, resulting in decreased substrate breakdown and little or no energy production. This is a problem, particularly in rural Africa where communities heavily rely on AD for energy. Therefore, cold-tolerant strains provide good opportunities for microbial-mediated psychrophilic AD (PAD) or bioaugmentation, which will help overcome the problems associated with process performance during AD in cold seasons [6]. A study by Rajagopal et al. [5] showed a methane percentage increase within the range of 64%–69%, indicating the efficiency of PAD of food waste at 20°C. These methane percentages are comparable to those obtained under mesophilic/thermophilic conditions.

Apart from its impact on AD, low temperatures can also negatively affect beneficial soil microbiota, which ultimately affects agricultural productivity. Hence, when considering AD bioaugmenting agents, it is also important to investigate if they are beneficial to crops and can thrive in winter for continuous agricultural activities. Digestate represents a unique habitat with a diversity of metabolically active microbial communities, both good and bad. However, more attention has been previously paid to pathogenic components due to the propensity of certain feedstock (animal manure) to harbor pathogens [14, 15]. Surprisingly, depending on factors such as digestion temperature, retention time, and contamination levels of the feedstock, the AD process has been shown to reduce pathogenic strains and enrich beneficial microorganisms [15, 16]. This caused a shift in thinking about the useful microorganisms in digestate, with more researchers now investigating plant growth-promoting bacteria (PGPB) contained in organic soil amendments, such as digestate, to assess their potential in agriculture [15–17].

Generally, PGPB stimulate plant growth by mobilizing nutrients in the soil, producing plant growth regulators, protecting plants from phytopathogens, and improving soil structure. Analysis of the PGPB in digestate is required to assess its agronomic potential based on its PGPB composition. To date, although reports indicating the presence of PGPB in digested residue are available, these studies have focused on mesophilic and thermophilic digestate [17, 18]. However, little is known about cold-tolerant PGPB in psychrophilic digesters. Information about cold-active PGPB present in psychrophilic digestate will provide new insights into its potential to improve crop yields after application in agricultural soils, particularly in cold areas.

Therefore, the study aimed to screen for cultivable and facultative anaerobic, psychrotrophs with plant growth-promoting (PGP) attributes in digestate sourced from low-temperature AD. An in vitro evaluation of PGP capabilities of the isolates was conducted to explore the potential of digestate as an organic soil ameliorant for sustainable agriculture. Furthermore, phenotypic characterization using Biolog's carbon utilization assay was performed to elucidate the metabolic capabilities and ecological roles played by the isolates. Molecular characterization of cold-tolerant PGPB isolates was also achieved through 16S rRNA gene sequencing. The study is one of the first investigations to report the characterization of bacterial isolates from PAD.

## 2. Methods and Materials

### 2.1. Sample Collection

Fresh cow dung (CD) samples were collected at the experimental farm of the Agricultural Research Council (Animal Production Institute), Pretoria (−25.8990023 S 28.2152523 E). Samples were placed in a cooler box and transported to the biogas laboratory at Agricultural Research Council–Soil, Climate and Water (ARC-SCW). All samples were immediately stored at 4°C until analysis (within a week), but those for molecular studies were stored at −20°C and −80°C.

### 2.2. Batch Culture Experiments and Acclimation

Acclimation assays were performed during batch experiments to enrich and select robust and cold-tolerant strains in low-temperature digestate, as described previously [19, 20]. The batch experiment was performed in airtight Schott bottles with a total and working volume of 500 and 200 mL, respectively. The CD was thoroughly mixed with distilled water in a 2-L Erlenmeyer flask to obtain a homogeneous mixture with a fixed total solid content of 10%. Subsequently, 200 mL of the prepared mixture was dispensed into each Schott bottle and tightly closed with a rubber septum. The bottles were incubated at 30°C (mesophilic condition) in a low-temperature IncoShake incubator (Model 355) (Labotec, Midrand, South Africa) at 90 rpm, and the batch culture was adapted by gradual decrement to 15°C from an initial temperature of 30°C. The acclimation was achieved in four stages as presented in Table 1. All experiments were performed in duplicate.

### 2.3. Bacterial Isolation From Psychrophilic Digestate

Cold-tolerant bacteria were isolated from psychrophilic anaerobic digestate obtained at the end (Day 42) of the acclimation period. Briefly, 1 g of the digested residue collected after a 42-day AD of CD was transferred to 9 mL of sterile distilled water and serially diluted up to 10^−8^. For each dilution, 100 *μ*L aliquots were spread on nutrient agar (Central Drug House (P) Ltd., New Delhi, India) plates and incubated at 15°C for 48 h. Following incubation, morphologically distinct bacterial colonies were selected and subcultured on nutrient agar plates several times until pure colonies were obtained. Pure isolates were preserved at −80°C in 20% glycerol for further study.

### 2.4. Determination of PGP Attributes

#### 2.4.1. Phosphate Solubilization

The ability of bacterial isolates to solubilize phosphate was determined on a Pikovskaya's agar plate (HiMedia Laboratories Pvt. Ltd., Maharashtra, India) [21]. Bacterial strains were spotted on Pikovskaya's agar plate and incubated at 15°C for 3–7 days. The isolates, which produced a halo zone around the colony, were determined as having the ability to solubilize phosphate.

#### 2.4.2. Atmospheric Nitrogen Fixation

The potential ability of the isolates to fix atmospheric nitrogen was assayed on Burk's nitrogen-free medium (Central Drug House (P) Ltd., New Delhi, India), which can be utilized for isolation of free-living, nitrogen-fixing bacteria [22, 23]. The medium contained in a liter is as follows: 0.2 g MgSO_4_, 0.80 g K_2_HPO_4_, 0.2 g KH_2_PO_4_, 0.130 g CaSO_4_, 0.00145 g FeCl_3_, 0.000253 g Na_2_MoO_4_, and 20 g sucrose. Bacterial isolates were inoculated in Burk's medium and incubated at 15°C for 3–7 days. The isolates that were able to grow after incubation, as revealed by the turbidity of the medium, were considered potential nitrogen fixing strains.

#### 2.4.3. Indole Acetic Acid (IAA) Production

The bacterial isolates were assessed for their ability to produce IAA as previously described by Khalid et al. [24], with slight modifications. Salkowski reagent was prepared by mixing 0.5 M FeCl_3_ and 35% HClO_4_ (ACS reagent grade, Merck KGaA, Darmstadt, Germany). Bacterial isolates were cultured in LB medium (Liofilchem S.R.L., Teramo, Italy) supplemented with 1% L-tryptophan (Sigma-Aldrich, Missouri, United States) in a rotary shaker at 150 rpm for 48 h at 15°C. The resulting cultures were centrifuged at 10,000 rpm for 10 min at 4°C. One milliliter of the supernatant was mixed with 2 mL of Salkowski reagent and incubated in the dark for 30 min at room temperature. A change in the color of the reagent from yellow to pink indicated IAA production.

#### 2.4.4. Siderophore Production

The ability of the isolates to produce siderophores was determined by the universal Chrome Azurol S (CAS) assay by Schwyn and Neiland [25]. The following analytical research grade chemicals were obtained: FeCl_3_; CAS, CTAB, casamino acids, quinosol, PIPES, and NaOH (Sigma-Aldrich, Missouri, United States); D-glucose (MINEMA Chemicals, Gauteng, South Africa); NH_4_Cl, NaCl, and chloroform (Rochelle Chemicals & Lab Equipment cc, Gauteng, South Africa); bacteriological agar (Biolab Diagnostics Laboratory Inc., Budapest, Hungary); and HCl and KH_2_PO_4_ (Labochem S.A., Gerakas, Greece). The CAS agar plates were prepared as per the method described by Louden et al. [26]. Isolates were spot inoculated on the prepared CAS agar plates in triplicate, while a noninoculated plate served as a control. Subsequently, plates were incubated at 15°C for 5–7 days and observed daily for the formation of a yellow-orange halo around the bacterial colonies, which would indicate siderophore production.

#### 2.4.5. Hydrolytic Enzyme Production—Cellulase Production

Cellulase activity of the bacterial isolates was tested according to the carboxymethyl cellulose (CMC)–Congo red agar staining method [27], with slight modifications. The media of the agar plates was composed of analytical research grade chemicals as follows: 0.05% KH_2_PO_4_, 0.1% K_2_HPO_4_ (Labochem S.A., Gerakas, Greece), 0.05% MgSO_4_ (Sigma-Aldrich, Missouri, United States), 0.05% KCl (Sigma-Aldrich, Missouri, United States), 0.2% CMC (HiMedia Laboratories Pvt. Ltd., Pennsylvania, United States), 0.02% peptone (Oxoid Ltd., Hampshire, United Kingdom), and 1.7% agar. Each colony was spot inoculated on an agar plate and incubated at 15°C for 48 h. After incubation, the plates were flooded with 0.1% Congo red (Sigma-Aldrich, Missouri, United States) for 15–20 min and then with 1 M NaCl for 15–20 min. A yellow halo against a red background of the Congo red stain indicated cellulase production by isolates.

#### 2.4.6. Hydrolytic Enzyme Production—Proteolytic Activity

All the isolates were screened for proteolytic activity in LB agar (Condalab, Madrid, Spain) supplemented with 1% skim milk (Oxoid Ltd., Hampshire, United Kingdom) [28]. Bacterial isolates were spot inoculated on skim milk agar plates, and plates were incubated for 5–7 days at 15°C. Proteolysis resulted in the formation of a clear zone around the bacterial colonies; the diameter of this clear zone was measured for proteolytic activity quantification.

#### 2.4.7. Biofilm Formation

The microtiter plate assay described by O'Toole and George [29] was slightly modified and employed for the detection of biofilm production by each bacterial isolate. Briefly, each strain was grown in 10 mL of LB medium and incubated at 15°C for 24 h. Overnight culture was adjusted to an OD_600_ of 0.1 by diluting each culture with fresh LB medium based on growth. Then, 1:100 dilution of standardized culture was prepared in a fresh assay medium. Prepared cultures were vortexed, and 100 *μ*L was aliquoted into a flat-bottom 96-well polystyrene microtiter plate. Blank wells containing 100 *μ*L of uninoculated growth medium were used as controls. Plates were covered and incubated at 15°C for 48 h. Following incubation, planktonic cells were removed by inverting the plates over a waste container. Thereafter, the plates were rinsed three times in sterile water and air-dried for 45 min. The microtiter plate wells were stained with 100 *μ*L of 0.1% crystal violet (CV) solution (Sigma-Aldrich, Missouri, United States) and incubated at room temperature for 45 min. The plates were inverted and gently shaken to remove the stain and rinsed three times in sterile distilled water. Subsequently, 150 *μ*L of acetic acid (30%) (ACS reagent grade, Sigma-Aldrich, Missouri, United States) was added to each well and pipetted up and down to ensure adequate solubilization of the biofilm. Then, 125 *μ*L of solubilized biofilm was transferred to a new microtiter plate, and its concentration was determined by measuring OD at 550 nm using a Synergy HTX Microplate Reader (BioTek, Winooski, Vermont, United States). All experiments, including the blanks, were performed in triplicate.

#### 2.4.8. Motility

The motility of the isolates was determined using sulfide indole and motility (SIM) medium based on a protocol published by Shields and Cathcart [30]. The SIM medium (Oxoid Ltd., Hampshire, United Kingdom) was composed of tryptone (20 g/L), peptone (6.1 g/L), ferrous ammonium sulphate (0.2 g/L), sodium thiosulphate (0.2 g/L), and agar (3.5 g/L) and prepared according to the manufacturer's instructions. Using a sterile needle, a well-isolated colony of each bacterial isolate was picked and stab-inoculated into sterile SIM medium in a test tube without touching the bottom of the tube. After inoculation, the needle was removed from the medium following the original stab line to prevent lateral movement of the needle that could produce confusing results. Inoculated SIM test tubes were then incubated for 48 h at 15°C. The motility in the isolates was confirmed by the diffuse spread of growth beyond the stab line of inoculation.

#### 2.4.9. Biolog Carbon Utilization Assay

Carbon substrate utilization by isolates was tested using the Biolog Phenotype MicroArray PM1 (Biolog Inc., Hayward, California, United States) microtiter plates according to the procedures developed by the manufacturer. The PM1 assay consists of 96-well microtiter plates with 95 wells prefilled with different carbon sources, and an additional well contains water as the negative control. Briefly, each bacterial isolate was cultivated on Biolog Universal Growth (BUG) agar for 24 h at 15°C to obtain isolated colonies. The cells were then harvested from the BUG agar plate using a sterile swab and transferred into inoculating fluid (IF) to achieve 42% transmittance (T). To obtain a cell suspension at the final cell density of 85% T, 15 mL of the 42% T cell suspension was mixed with 75 mL of an IF-dye solution. Subsequently, 100 *μ*L of the final cell suspension was inoculated into each well of the PM1 microplate. Thereafter, each plate was wrapped with parafilm to minimize evaporation and incubated at 15°C for 48 h. Following incubation, carbon usage by each isolate was measured by reading and recording the color change in individual wells of the Biolog plate with a Synergy HTX Microplate Reader (BioTek, Winooski, Vermont, United States). A change from colorless to purple denoted the ability of the cells to metabolize the incorporated substrate in the well.

### 2.5. Growth Curve Determination

The growth assay of the isolates was conducted in triplicate using a method described by Zubair et al. [31] with some modifications. Bacterial strains were grown in LB medium and incubated for 48 h at 15°C. The optical density of the culture was measured at 600 nm (OD_600_) and adjusted to a uniform cell density, and then, 20 mL of each culture was incubated at 15°C with agitation at 100 rpm. The growth of the isolates was determined by periodically measuring absorbance at 600 nm of 200 *μ*L aliquots of each culture over 112 h using a Synergy HTX Microplate Reader (BioTek, Winooski, Vermont, United States).

### 2.6. Molecular Identification of Isolates

#### 2.6.1. DNA Extraction and Quantification

Genomic DNA of isolates was extracted using a Quick-DNA fungal/bacterial kit D6005 (Zymo Research, United States) according to the manufacturer's guidelines. The quality and quantity of extracted DNA were determined using 1% agarose gel electrophoresis and a Qubit 2.0 fluorometer (Invitrogen, California, United States), respectively.

#### 2.6.2. 16S rRNA Amplification and Sequencing

The polymerase chain reaction (PCR) of the extracted genomic DNA was performed in a Bio-Rad T100 PCR thermocycler (California, United States) as previously described [32]. Each 25 *μ*L reaction mix consisted of 12.5 *μ*L of OneTaq 2X Master Mix with Standard Buffer (New England Biolabs, Massachusetts, United States), 50 ng/*μ*L of DNA template, 0.2 *μ*M each of forward (8F: 5⁣′-*AGAGTTTGATCCTGGCTCAG*-3⁣′) and reverse (1392R: 5⁣′-*ACGGGCGGTGTGTAC*-3⁣′) primers [33], and sterile nuclease-free water. A reaction mixture without a DNA template was used as the negative control. The PCR conditions were as follows: initial denaturation step at 94°C for 30 s, 35 cycles of 30-s denaturation at 94°C, annealing at 55°C for 60 s, elongation at 68°C for 60 s, and a final elongation step at 68°C for 5 min. Thereafter, the PCR products were sent to Inqaba Biotechnical Industries (Pty) Ltd., Pretoria, South Africa, for sequencing with the ABI 3500xL Genetic Analyzer (Thermo Fisher Scientific Inc., Massachusetts, USA). The obtained sequences were checked for quality and assembled using Geneious Prime 2023 (https://www.geneious.com). All sequences were compared to the GenBank nucleotide data library using the basic local alignment search tool (BLAST) software [34] on the National Centre for Biotechnology Information (NCBI) platform (http://www.ncbi.nlm.nih.gov).

#### 2.6.3. Phylogenetic Analyses of Isolates

For each sequence data, sequence homology was used for the preliminary identification of isolates using BLAST. The mothur software pipeline was used to cluster sequences of isolates with the most outstanding abilities into operational taxonomic units (OTUs) at a sequence similarity of ≥ 99%. Phylogenic reconstruction of the sequences was done by multiple sequence alignments, using MAFFT [35], of sequences of the seven isolates with closely related sequences from GenBank, as well as an outgroup sequence of *Clostridium perfringens*. The aligned multiple sequences were edited and imported to Geneious Prime 2023 (https://www.geneious.com) in which phylogenetic relationship was inferred using the neighbor-joining [36] tree building method and the genetic distance model of Tamura-Nei [37] with 1000 bootstrap replications. The 16S rRNA sequences were submitted to NCBI GenBank and assigned accession numbers OQ132787–OQ132793.

### 2.7. Statistical Analyses

Statistical analyses of all replicate data were conducted using a one-way analysis of variance (ANOVA) followed by Tukey's post hoc test (*p* ≤ 0.05) using RStudio software (2024.04.2 Build 764) (RStudio Team, Massachusetts, United States). Different letters were used to represent significant differences in performance between isolates.

## 3. Results

### 3.1. Isolation and PGP Traits of Bacterial Isolates

A total of 20 facultative, cold-tolerant bacteria strains were isolated from digestate obtained from PAD. The isolates displayed a variety of morphological features such as elevation, color, shape, margin, and size, as shown in Table 2. All strains grew well after incubation at 15°C for 48 h. All isolates were screened at 15°C on specific media to assess their in vitro plant growth promotion traits. Eventually, the seven most promising psychrophilic PGP isolates designated as A3-1, A3-3, A3-4, B5-1, B5-2, B5-3, and B5-5 were selected for further analyses (Table 3).

In the present study, of the 20 cold-tolerant isolates, 12 (60%) were able to solubilize phosphate from insoluble compounds in a solid medium at 15°C. Nine (45%) isolates exhibited N fixation activity in a N-free medium, and six (30%) isolates showed IAA production. However, unlike other PGP attributes, siderophore production was less common among isolates, with only two (10%) strains showing this trait on CAS agar plates. Furthermore, hydrolytic enzyme production also varied with cellulase production observed as a common trait since all isolates produced varying levels of cellulase ranging from 3.3 to 15.3 mm activity diameter. Isolates A3-1, B5-1, and B5-5 displayed maximum cellulolytic activity with activity diameters of 13, 13 and 15.3 mm, respectively, whereas only two of the bacterial isolates (B5-5 and B5-2) produced protease, of which B5-5 exhibited maximum proteolytic activity with an activity diameter of approximately 11 mm (Table 3).

### 3.2. Molecular Identification of Most Promising Isolates

The seven most promising psychrophilic PGP isolates designated as A3-1, A3-3, A3-4, B5-1, B5-2, B5-3, and B5-5 were selected and identified using molecular methods (presented in italics in Table 3). PCR amplification of 16S rRNA gene yielded a single amplicon of approximately 1500 bp for each of the seven cold-tolerant isolates. Each isolate sequence had an identity with homologous sequences within the GenBank library. In addition, taxonomic assignment of the seven PGP strains revealed that isolates belong to the phylum Proteobacteria (Table 4).

Comparative sequence analysis of BLASTn search on NCBI revealed that four out of seven isolates designated as A3-3, A3-4, B5-1, and B5-3 belonging to the genus *Acinetobacter* were identified as *Acinetobacter iwoffi.* On the other hand, two isolates designated as A3-1 and B5-2 belonging to genus *Comamonas* were identified as *Comamonas* sp., and the remaining isolate designated as B5-5 belonging to genus *Pseudomon*as was identified as *Pseudomonas* sp. Together, these results suggest that A3-3, A3-4, B5-1, and B5-3, as well as A3-1 and B5-2 are likely to be different strains of the same species. Henceforth, strains will be referred to as the “strains per OTU” names assigned in Table 4.

### 3.3. Colonization Phenotype—Motility and Biofilm Formation

**Figure 1:**
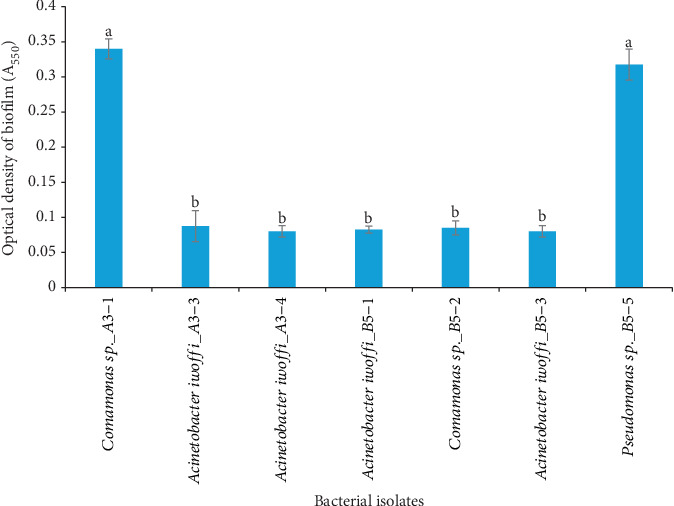
Biofilm formation recorded at 550 nm of the seven most promising bacterial isolates at 15°C. Bars indicate standard deviation (*n* = 3), and different letters above bars indicate significant differences at *p* ≤ 0.05 according to a one-way ANOVA and Tukey's test.

Each of the seven selected isolates possessed at least four PGP attributes, and their evaluation for motility and biofilm production revealed that only three, that is, *Comamonas* sp._A3-1, *Comamonas* sp._B5-2, and *Pseudomonas* sp._B5-5, exhibited motility when cultured in sulphide motility medium at 15°C. On the other hand, all isolates formed biofilm that ranged from 0.08 (OD_550_) to 0.34 (OD_550_) (Figure 1), but over 72% produced less than 0.09. Isolates *Comamonas* sp._A3-1 and *Pseudomonas* sp._B5-5 produced higher amounts at 0.34 (OD_550_) and 0.31 (OD_550_), respectively (Figure 1).

### 3.4. Growth Curve Determination

**Figure 2:**
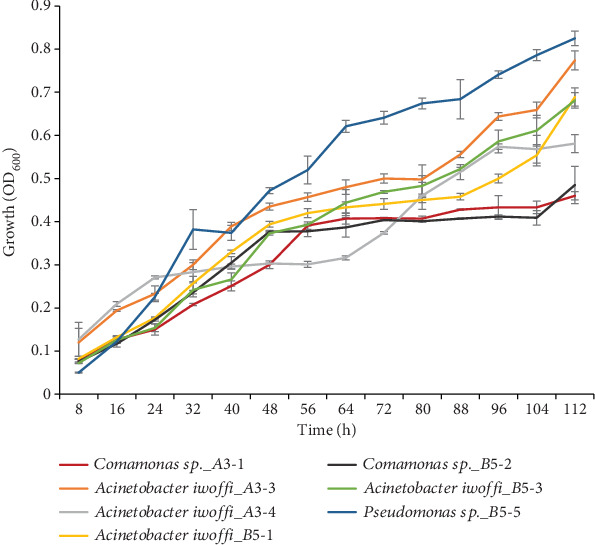
Growth curves recorded at 600 nm of the seven most promising isolates at 15°C.

The growth curves of the selected bacterial isolates showed that they are quite adapted to the temperature of incubation (15°C) as growth started immediately but at a relatively slow rate as revealed by the slope of the curve (Figure 2). Although the growth of strain *Pseudomonas* sp._B5-5 was initially slow, it later surpassed others and had the fastest growth after 112 h of incubation at 15°C.

### 3.5. Carbon Utilization Profiles of Isolates

**Figure 3:**
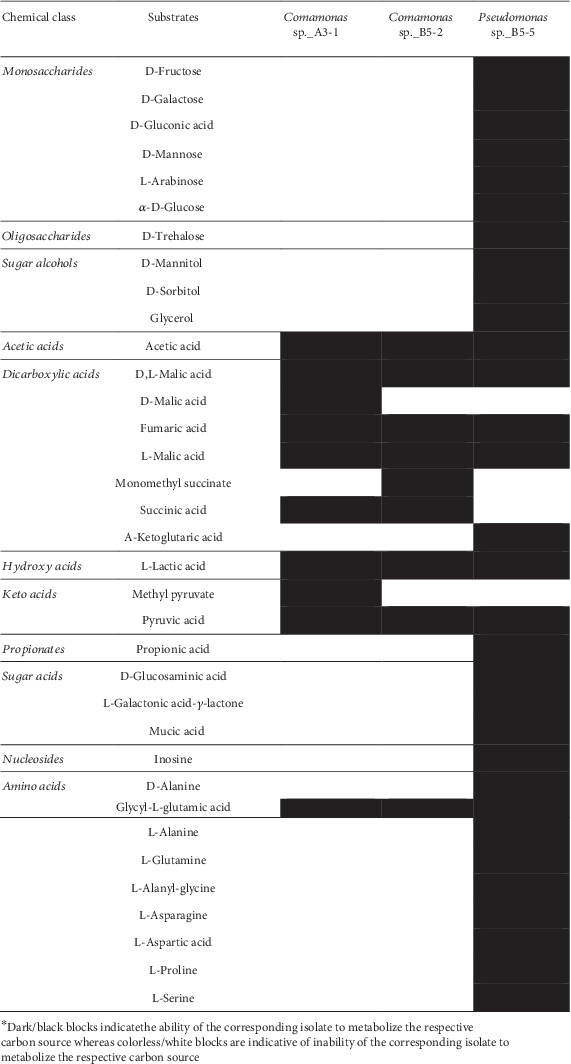
Biolog carbon utilization profiles of isolates that showed observable growth/respiration in Biolog media. Dark/black blocks indicate the ability of the corresponding isolate to metabolize the respective carbon source, whereas colorless/white blocks are indicative of the inability of the corresponding isolate to metabolize the respective carbon source.

The carbon utilization profiles for all isolates at 15°C are summarized in Figure 3, which revealed variability in carbon usage. For example, four strains, *Acinetobacter iwoffi_*A3-3, *Acinetobacter iwoffi_*A3-4, *Acinetobacter iwoffi_*B5-1, and *Acinetobacter iwoffi_*B5-3, did not show observable growth or respiration in Biolog media under the conditions required for the assays and therefore were not assessed for carbon utilization. Whereas isolates *Comamonas* sp._A3-1 and *Comamonas* sp._B5-2 utilized 10 and 9 carbon sources, respectively, and demonstrated growth on VFAs and amino acids. On the other hand, isolate *Pseudomonas* sp._B5-5 possessed the highest metabolic versatility by utilizing 31 (33%) of the 95 carbon sources tested, including dicarboxylic acids, various nucleotides, and some amino acids.

## 4. Discussion

The study of microorganisms in PAD systems is an emerging research area in recent years, since there is an increasing interest in PAD technology as an approach for organic matter (OM) degradation under low temperatures [6, 9, 13]. Psychrophilic anaerobic digesters constitute an ideal source of cold-tolerant microorganisms [38]. Cold-tolerant microbes counteract the negative effects of low temperatures, colonize, and flourish under otherwise harsh conditions. Consequently, this unique group of microorganisms are of considerable importance during PAD and possess enormous potential in agriculture and other industries.

In this study, we described for the first time cultivable cold-tolerant bacteria in psychrophilic digestate and their biotechnological potentials. Our exploration of cultivable strains in psychrophilic digestate revealed various bacterial isolates, with numerous capabilities. The isolates belong to the phylum Proteobacteria which has also been reported as plant growth promoters and found in cold environments. The phylum is very versatile with flexibility and adaptation to various environments [39]. The *Pseudomonas* bacteria are generally recognized as ecologically successful bacteria, associated with many extreme habitats due to their versatile metabolism and the ability to tolerate various biotic and abiotic stresses, which explains their presence across a range of cold ecosystems [38]. In this study, the genus *Pseudomonas* was detected with a low abundance consisting of only one isolate (*Pseudomonas* sp._B5-5). The low occurrence of *Pseudomonas* in this work possibly reflected the influence of ecosystem variation on the bacterial distribution and diversity in different ecological niches [40]. The most abundant genus obtained in this study, *Acinetobacter*, are well-known plant growth promoters which have gained agricultural prominence worldwide. They are known to solubilize phosphate, potassium, and zinc. They also produce IAA, siderophores, gibberellins, antibiotics, and biosurfactants making them important PGPB [41]. *Acinetobacter* were also reported in cold-adapted environments providing cold alleviation to wheat plants [42].

The ability of bacterial isolates to fix atmospheric nitrogen is one of the essential traits through which plant growth promotion is evaluated. Biological N fixation involves the transformation of atmospheric N into plant-accessible forms by a specialized group of microorganisms [43]. Approximately 50% of the psychrophilic isolates characterized in this study, including *Acinetobacter* spp., *Comamonas* sp., and *Pseudomonas* sp., could fix atmospheric nitrogen. In agreement with our results, nitrogen fixation has been reported for cold-tolerant bacterial species of *Arthrobacter*, *Bacillus*, *Providencia*, *Pseudomonas*, *Acinetobacter*, and *Stenotrophomonas* [44].

Phosphate solubilization is another important PGP trait [45]. Phosphate-solubilizing bacteria promote phosphate availability by solubilizing precipitated and fixed phosphorus in soils and supplying plant roots with soluble P. As demonstrated in this study, 60% of the isolates exhibited phosphate solubilization abilities at a low temperature (15°C). Similarly, *Pseudomonas* strains with the ability to solubilize phosphate have been isolated from other cold environments [46, 47].

The biosynthesis of phytohormones such as IAA by PGPB facilitates plant–microbe interactions and promotes plant growth by improving root development for improved mineral and nutrient uptake [48]. In this study, some of the isolates were IAA producers, which is corroborated by several other studies evaluating psychrotolerant isolates [38, 39, 49].

The role of microbial siderophores in plant iron nutrition is well documented. These iron chelators are able to solubilize organic and inorganic iron complexes, consequently increasing iron availability in soil for plant uptake [50]. Thus, siderophore producers primarily promote plant growth by improving soil fertility and enhancing iron absorption by plants [51]. Additionally, by depriving other soil microbes, particularly pathogens of essential iron, siderophore-producing bacteria can exhibit antagonist effects against a range of phytopathogens [52]. Unlike other PGP traits that were widespread among bacterial isolates, siderophore production was limited to a few isolates (B5-2 and B5-5) identified as *Comamona*s sp. and *Pseudomonas* sp.

Besides direct promotion of plant growth through the fixation of atmospheric N, solubilization of inorganic phosphate, and production of IAA and siderophores, beneficial microorganisms also indirectly protect plants against many phytopathogens via the secretion of cell-wall degrading hydrolytic enzymes [53]. Bacteria-produced hydrolytic enzymes, including chitinase, cellulase, and protease, inhibit the growth of plant pathogens via hydrolysis of their cell wall, proteins, and DNA [49]. PGPB with the ability to synthesize at least one of these extracellular hydrolytic enzymes have been shown to have significant efficacy in the control of disease of important crops and are promising alternatives to agrochemicals [49]. Among the tested isolates in the present study, only *Acinetobacter iwoffi*_B5-3 and *Pseudomonas* sp._B5-5 exhibited proteolytic activity; on the contrary, cellulase production was a common trait, as all isolates produced varying levels of cellulase ranging from 3.3 to 15.3 mm activity diameter. The dominance of cellulose-degrading bacteria could be attributed to the nature of the substrate used for AD (CD) which is generally rich in complex carbohydrates including cellulose, hemicellulose, and lignin [54]. The high content of complex sugars in CD could have selected bacteria with a higher fitness on a cellulose-rich diet given that substrate is an important factor shaping the microbial community in an ecosystem [55]. In summary, the selected digestate PGP isolates demonstrated efficient in vitro plant growth promotion characteristics at low temperatures.

To exert the expected beneficial effects under field conditions, PGPB should be able to survive and successfully colonize the plant roots and rhizosphere [56]. Factors related to bacteria movement toward chemical signals and root exudates as well as those involved in adherence and colonization of root surface, such as bacterial motility and biofilm production, play a significant role in their colonization outcome. Thus, bacterial motility and biofilm formation should be an integral screening criteria for PGPB candidates [57]. Motility has an important implication in rhizosphere colonization as mutants with reduced motility are poor competitors and lack efficient adhesion to plant roots [58]. On the contrary, hypermotile variants that are more competitive are selected in the root zone. Based on the 16S rRNA sequences, the motile strains in this study were identified as *Pseudomonas* and *Comamonas* spp. This is in agreement with previous studies that described bacterial motility among the same genera of bacteria [59, 60].

Another important colonization determinant is biofilm formation. Microbial biofilms consist of microbial communities embedded in extracellular matrices that provide structure and protection against unfavorable environmental conditions [61]. Biofilm formation within the root zone promotes direct interaction between beneficial microorganisms and plants thereby facilitating nutrient exchange between counterparts and a direct supply of phytohormones and antimicrobial compounds [57]. Further, PGP properties are higher in biofilms than in planktonic cells since biofilms enable bacteria to maintain an elevated number of cells for enhanced beneficial interactions in the rhizosphere. *Pseudomonas* is a well-studied biofilm producer that has proven to be an efficient colonizer of a variety of crops including peas and tomatoes [52, 62]. According to our results, *Pseudomonas* was also identified as an efficient biofilm producer together with *Acinetobacter* and *Comamonas* sp. The biofilm-forming capacity of our isolates revealed an interesting association with their motility trait. With the exception of *Comamonas* sp._B5-2, which produced minimal biofilm, all other motile isolates were good biofilm producers. This seems to support the assertion that motility and the presence of cellular structures such as flagella and pili may play a role in the formation of biofilm matrix [52]. The finding of motile and biofilm-forming strains in digestate indicates that these bacteria could be useful for successful colonization and adhesion to plant roots for increased plant nutrition and effective plant growth promotion.

With the aim of elucidating the putative metabolic and ecological roles of cold-tolerant isolates in PAD, we used the Biolog PM1 assay to characterize their degradation capabilities. Biolog analysis of isolates from the current study revealed that three carbon utilization patterns and isolates were accordingly assigned into three categories. The first group showed no metabolic activity and was shared by *Acinetobacter* isolates (*Acinetobacter iwoffi*_A3-3, *Acinetobacter iwoffi*_A3-4, *Acinetobacter iwoffi*_B5-1, and *Acinetobacter iwoffi*_B5-3) characterized by the absence of observable growth on Biolog plates. The second group comprising of *Comamonas* isolates (*Comamonas* sp._A3-1 and *Comamonas* sp._B5-2) displayed lowered metabolic versatility as compared to *Pseudomonas* sp._B5-5, the only member of the third group with diverse metabolism. As previously reported in this study, the same isolate, *Pseudomonas* sp._B5-5, produced different hydrolytic enzymes such as cellulases and proteases that are often associated with the degradation of complex polysaccharides [63]. Therefore, *Pseudomonas* sp._B5-5 presents enormous potential as a bioaugmentation strain to promote the hydrolysis of numerous poorly degradable lignocellulose wastes during PAD. Acetic acid is among the principal organic acids affiliated with AD irrespective of the temperature of digestion [64]. Consequently, a number of these organic acids are preferred by our isolate. The described utilization patterns have provided new insights into the diversity of carbon utilized by PAD isolates. In *Comamonas* spp., analysis of the core genome revealed conserved acetate and pyruvate pathways and a lack of key genes for glucose phosphorylation [65]. Consistent with core genome analysis, *Comamona*s strains obtained in our study grew well on various amino acids and organic acids, but rarely catabolize pentoses or hexoses. Similar to the observed lack of growth among some isolates, analysis of facultative anaerobes from coal seams reported the absence of growth in some isolates on Biolog plates [66]. Our results showed that Biolog PM1 plates are useful tools to elucidate cellular phenotypes among bacteria and provide evidence for the hypothesis that it is a high-throughput technique for characterizing cellular phenotypes since carbon utilization profiles correlate with 16S rRNA identification.

Overall, this study highlights the residue of PAD as a reservoir of cultivable and cold-tolerant bacterial strains that exhibit remarkable PGP and AD-enhancing properties. Therefore, they represent an invaluable resource for further identification of plant-beneficial and possible AD-promoting microorganisms. Cold-tolerant and hydrolytic bacteria, including different genera such as *Pseudomonas* and *Acinetobacter*, have a huge potential as bioaugmentation candidates for optimization of PAD. Furthermore, digestate enriched with cold-adapted and plant-beneficial bacteria may have applications in various agricultural processes during cold periods. Nevertheless, future work could seek to validate the observed beneficial traits in vivo under controlled and field conditions as well as genotypically through whole genome sequencing. Moreover, further work should be conducted to evaluate the isolates' propensity to act as a pathogen to plants, humans, or animals. Finally, the data provided in this study may pave way for the development of a model for prospecting bacteria from psychrophilic anaerobic digestate for potential agricultural applications in various parts of the world that experience cold temperatures that impact negatively on plant growth.

## Figures and Tables

**Table 1:** Stepwise acclimation of batch culture anaerobic digestion.

**Stage**	**Temperature (°C)**	**Time (days)**
1	30	0–7
2	25	8–14
3	20	15–28
4	15	29–42

**Table 2:** Morphological features of isolates.

**Number of isolates**	**Size**	**Elevation**	**Margin**	**Color**	**Shape**
5	Large	Raised	Smooth	Yellow	Round
6	Small/pin-point	Raised	Smooth	Cream	Round
9	Large	Raised	Smooth	Cream	Round

**Table 3:** Plant growth-promoting and biocontrol potential of isolates with the seven most promising isolates presented in italics.

**Isolate**	**Phosphate solubilization (activity diameter [mm])**	**Nitrogen fixation**	**IAA production**	**Siderophore production (activity diameter [mm])**	**Cellulase production (activity diameter [mm])**	**Protease production (activity diameter [mm])**
*A3-1*	7 ± 0^ab^	*G*	*+*	−	13 ± 2^ab^	−
A3-2	−	G	−	−	8.3 ± 1^abc^	−
*A3-3*	1 ± 0^d^	*G*	*+*	−	9.3 ± 2^abc^	−
*A3-4*	6.3 ± 1.5^bc^	*G*	*+*	−	3.3 ± 0.5^c^	−
A3-5	−	N/G	−	−	7 ± 2^abc^	−
A4-1	8.3 ± 1.5^a^	N/G	−	−	8 ± 2^abc^	−
A4-2	1 ± 0^d^	N/G	−	−	5.3 ± 1^bc^	−
A4-3	−	G	−	−	5.5 ± 2^bc^	−
A4-4	−	G	−	−	7.6 ± 2^abc^	−
A4-5	−	N/G	−	−	3 ± 0.5^c^	−
*B5-1*	6 ± 1^bc^	*G*	*+*	−	13 ± 1.1^bc^	−
*B5-2*	1 ± 0^d^	*N/G*	*+*	5 ± 1^b^	11 ± 2.6^abc^	−
*B5-3*	6.3 ± 0.6^bc^	*G*	−	−	7.3 ± 1.5^abc^	5.3 ± 1.5^b^
B5-4	−	N/G	−	−	6.6 ± 1.5^abc^	−
*B5-5*	5 ± 1^c^	*G*	−	14 ± 1^a^	15.3 ± 4.05^a^	11.3 ± 2.9^a^
B6-1	6 ± 1^bc^	N/G	−	−	4.4 ± 1^c^	−
B6-2	1 ± 0^d^	N/G	−	−	5.2 ± 2^bc^	−
B6-3	−	N/G	+	−	4.3 ± 0.6^c^	−
B6-4	−	N/G	−	−	4.5 ± 1.1^c^	−
B6-5	1 ± 0^d^	N/G	−	−	7.4 ± 1.1^abc^	−

*Note:* The symbol “+” represents a positive reaction or the presence of the trait of interest, while the symbol “−” shows a negative reaction or the absence of the particular trait. “G” indicates that growth was observed, whereas “N/G” indicates the absence of growth. All values are mean ± standard deviation of three replications. Different letters, in superscript, indicate significant differences at *p* ≤ 0.05 according to a one-way ANOVA and Tukey's test.

**Table 4:** Summary of OTUs (computed with mothur software) in relation to the genera and phyla.

**OTU**	**Representative isolate**	**Strains per OTU**	**Genus**	**Phylum**
OTU1	*Acinetobacter iwoffi*_B5-3	*Acinetobacter iwoffi_*A3-3, *Acinetobacter iwoffi_*A3-4, *Acinetobacter iwoffi*_B5-1, *Acinetobacter iwoffi_*B5-3	*Acinetobacter*	Proteobacteria
OTU2	*Comamonas* sp._B5-2	*Comamonas* sp._A3-1, *Comamonas* sp._B5-2	*Comamonas*	Proteobacteria
OTU3	*Pseudomonas* sp._B5-5	*Pseudomonas* sp._B5-5	*Pseudomonas*	Proteobacteria

## Data Availability

The sequence data that supports the findings of this study is openly available in GenBank under accession numbers OQ132787–OQ132793.
